# Oral Surgery

**Published:** 1897-02

**Authors:** Edmund W. Roughton


ARTICLE VI.

### ORAL SURGERY.

BY EDMUND W. ROUGHTON, B. S., M. D. (LOND.), F. R. C. S. ENG.

Diseases of the Gums. The mucous membrane of the gums is liable to the same inflammatory affections as that lining the rest of the buccal cavities. These and tumors of the gum have already been described, but there are some other diseases which require mention.

*Hypertrophy of the Gums.* This may result from the irritation caused by badly fitting dentures or accumulations of tartar. In such cases the hypertrophy is not great, and it is very seldom that it is necessary to do anything more than remove the cause of irritation. But in children a peculiar variety of this disease is sometimes encountered. It commences at the time that the temporary teeth are being cut—between the ages of six months and two years. The gum increases in size so that eventually the teeth become almost completely hidden from view by large papillomatous or polypoid-looking projections of the same color as the normal gum; although in places they are soft, vascular and spongy-looking, they are mostly firm and fibrous to the touch. The disease usually affects the whole of the alveolar arch in both jaws, but may sometimes be limited to the incisor region. In some cases the overgrowth is sufficiently large to project from the mouth and to bulge out the cheeks. Mastication is considerably hampered.

Microscopic examination proves the growth to be a pure hypertrophy of the gum, chiefly the fibrous portion. In structure it consists of a dense stroma of interlacing fibers, containing much glandular tissue in its interstices and covered on its surface by a large and vascular papillæ. The growth appears to start from the periosteum around the necks of the teeth.

The subjects of this disease are often deficient mentally, Sometimes it occurs in several members of the same family.

*Treatment.* It will not suffice simply to pare away the hypertrophied tissue, as recurrence is pretty sure to follow. This is owing to the disease affecting the sockets of the teeth as well as the gum. To effect a permanent cure it is necessary to move the alveolar margin as well. The germs of the permanent teeth in the vicinity of the disease must be avoided as far as possible.

*Polypus of the gum* is the name given to a localized hypertrophy of a portion of gum usually between two teeth. It is produced by the irritation of a rough or carious tooth, tartar, or some portion of an artificial denture. In microscopic structure it resembles gum-tissue. Sometimes the gum encroaches upon the cavity of a carious tooth so as to simulate polypus of the pulp, but it may be distinguished from the latter by its greater sensitiveness and by its pedicle or base of attachment being between the teeth and not within the carious tooth.

*Treatment* consists in removing the source of irritation and snipping off the growth with scissors. Its base should be touched with nitrate of silver or with the electric cautery. Recurrence does not take place after effectual removal.

Diseases of the Floor of the Mouth:

*Ranula.* A ranula is a cyst under the tongue, usually on one side of the frænum. Different views have been held as to the nature of the cyst and its mode of formation. It was formerly thought that it was always the result of dilatation of Wharton's duct. Although some ranulæ may be due to this cause, the majority of them are of a different origin; the shape of the swelling is not that of a dilated duct of Wharton, nor is the submaxillary gland itself swollen as it is in cases of obstruction of the duct by a salivary calculus; moreover, it is sometimes possible to pass a fine probe along the duct for an inch or more by the side of the cyst. Ranula has also been attributed to dilatation of one of the ducts of the sublingual gland, but the shape of the swelling and the condition of the gland itself negative this view. It is now held that the disease is usually due to to the dilatation of the duct of the Blannin-Nuhn gland, a small mucous gland situated on the under surface of the tongue a little to one side of the middle line. Von Recklinghausen had the opportunity of dissecting a subject in whom there was a ranula, and discovered the remains of the Blandin-Nuhn gland projecting into the cavity of the cyst; he also found that the epithelium lining the ranula was similar to that of the gland.

The disease is nearly always very chronic; it causes no pain and may pass for a long time unnoticed. The only subjective symptoms which it produces are a slight discomfort in mastication and a sense of fulness under the tongue. When the mouth is opened and the tongue turned back a ranula is plainly visible as a smooth bulging swelling of a deep bluish color, tinged with pink, and more or less translucent; large tortuous vessels are often seen coursing upon its surface. It is usually very soft to the touch, but may feel tense; fluctuations can be easily detected.

*Treatment* usually adopted is to cut out a portion of the cyst wall with scissors; the fluid thus evacuated is viscid. A simple incision without removal of a portion of the cyst-wall is usually followed by a reaccumulation of the fluid. Some surgeons prefer to introduce a seton and leave it in position for a week. If the above method fail, a triangular flap of the cyst-wall should be cut and stretched back into the cavity of the cyst; this effectually prevents the cyst from closing before the cavity is filled up. Lastly, the cyst may be completely removed, but the proceeding is a difficult one.

*Dermoid Cysts.* These in the floor of the mouth are due to the folding in of a portion of the integument during the coalition of the two lateral halves during development, or to imperfect obliteration of the lingual duct. In this manner a cavity lined by skin is formed. The epithelial lining of the cyst wall is usually tough and fibrous; it produces a thick material resembling sebaceous matter and composed of cast off epithelial cells, oil, cholesterine, fatty debris and sometimes hairs. The cyst is situated either in the middle line between the two genio-hyo-glossus and the mylo-hyoid. It is usually single, but sometimes one is found on each side of the middle tine.

Although of congenital origin it is very seldom that dermoid cysts in the floor of the mouth are noticed before the age of fifteen or twenty. The subjective symptoms which they produce are of but slight importance, although more pronounced than those produced by ranula, owing to the more solid nature of the cyst contents; discomfort in eating and speaking and a sense of fulness are usually the only symptoms complained of.

The tumor projects both into the floor of the mouth and in the neck between the chin and the hyoid bone, forming a lump as large as a hen's egg or larger. The surface of the tumor is smooth and its outline rounded or elongated; the mucous membrane covering it is of a yellowish tint, and nct translucent as in ranula; pressure produces distinct pitting in some cases. Fluctuation can usually be obtained both inside the mouth and in the neck, but the feeling is much more doughy than in a case of ranula. The diagnosis is usually easily made. In case of doubt it may be cleared up by an exploratoiy puncture.

*Treatment.* The cyst may be incised through the mouth, and the cavity packed with gauze after evacuation of the contents. It is, however, more satisfactory to reremove the sac completely; if the tumor be small this can be easily done from within the mouth. If the tumor be a large one it must be removed through an incision below the jaw, either in the middle line of the neck or over the most prominent part of the tumor, the dissection is not difficult or dangerous.

*Salivary Calculus.* A salivary calculus not very uncommonly forms in the duct of the submaxillary gland. In size and shape it somewhat resembles a fragment of slate pencil. It consists of phosphat and carbonat of lime and phosphat of magnesia. It forms very slowly and may remain for years without causing any symptoms; but eventually inflammation is set up, the surrounding tissues become swollen and painful, and the duct more or less completely obstructed. As the result of this obstruction and of inflammation spreading backwards along the duct the submaxillary gland becomes enlarged.

When the interior of the mouth is examined the tongue is found somewhat swollen; the parts between the tongue and the floor of the mouth on the affected side are red, swollen and tender. On palpating beneath the jaw the submaxillary gland is found to be enlarged and hard, but not as a rule tender to the touch. The presence of the symptoms just enumerated should always lead to an examination of the submaxillary duct. On introducing a fine probe a rough and gritty mass will be felt, usually near the orifice, but sometimes further back.

*Treatment.* The calculus must be removed through an incision made directly over it. Great care should be taken not to break it, as any fragments left behind are difficult to remove and are apt to cause even greater irritation than the original concretion.—*British Journal of Dental Science.*

